# Vaccination Against Mumps, Aren’t We Late Already?

**DOI:** 10.31729/jnma.8731

**Published:** 2024-08-31

**Authors:** Sajal Twanabasu, Sushan Homagain, Sucharita Tuladhar, Sujina Maskey, Ashika Mahaseth, Bhusan Kumar Subedi, Dinesh Regmi

**Affiliations:** 1 Dhading Hospital, Dhadingbesi, Dhading, Nepal; 2 Patan Academy of Health Sciences, Lagankhel, Lalitpur, Nepal

**Keywords:** *encephalitis*, *mumps*, *Nepal*, *vaccine*, *virus*

## Abstract

Mumps is a highly contagious viral infection caused by paramyxovirus. It usually presents with fever and parotid gland swelling. It may be associated with complications like aseptic meningitis, encephalitis, oophoritis, orchitis, pancreatitis. The incidence of mumps infection is increasing in Nepal. This paper aims to advocate for the introduction of vaccination against mumps in the national immunization schedule.

## INTRODUCTION

Mumps is an acute viral infection caused by paramyxovirus. Fever and swelling of the parotid gland either unilateral or bilateral, headache, and myalgia are the predominant symptoms. Mumps is a childhood disease that mainly affects children between 5-9 years old although adolescents and adults can also be affected.^1^ Asymptomatic infections occur in 15 to 20% of the cases.^2^ Orchitis, oophoritis, pancreatitis, encephalitis, and aseptic meningitis are the common complications seen in mumps.^3^ There is no specific treatment for mumps. Vaccine is the only effective measure to reduce the mumps related social, clinical, and economic problems. This paper aims to advocate for the introduction of the mumps vaccine in the national immunization schedule of Nepal.

## PREVALENCE

Mumps is not a notifiable or reportable disease hence it is difficult to estimate the global incidence of mumps though it occurs globally.^4^ It has been hypothesized that the burden of mumps is around 100-1000 cases per one hundred thousand population with epidemic peaks every 2-5 years in countries without routine mumps vaccination.^5^ Hence, it is obvious that the mumps epidemic is more common in countries without routine immunization against mumps. Nepal has not included mumps-containing vaccines in the national immunization schedule and hence there are occasional outbreaks but the actual incidence is not known. Last year there was a nationwide epidemic of mumps. Nepal Pediatric Society (NEPAS) attempted to document this unprecedented surge of mumps using a Google questionnaire developed by the World Health Organization (WHO) where around 273 cases were reported in 3 months across the country.^6^ This seems an underestimation as all the cases were not reported. Dhading Hospital also saw a rise in mumps cases, which was evident from the records of the OPD register, where 124 cases were diagnosed clinically as mumps in 3 months from the 15^th^ of May 2023 to the 14^th^ of July 2023.

Keyal et al. also reported 113 mumps cases over a span of two years at Civil Hospital between November 2009 to October 2011.^7^ There is no doubt that the incidence of mumps is increasing in Nepal. With that, we are also risking our babies with the potential complications of mumps.

## WHY IS VACCINATION A CONCERN?

Mumps is a self-limited disease, complications can be frequently encountered. Death is rare (1/10,000) which is usually due to mumps encephalitis. Cerebrospinal fluid alterations are seen in more than 50% of cases among which 1-10% develop signs and symptoms of aseptic meningitis and 0.1% of encephalitis. Unilateral sensorineural deafness (5/100,000) is the most common long-term complication of mumps in children.^8^

Orchitis is seen in 20% of postpubertal males. Mumps orchitis is also a risk factor for testicular cancer.^9^ Oophoritis, pancreatitis, and mastitis are the other complications.^3,9^ Similarly, mumps virus infection during the first 12 weeks can lead to abortion. One study showed that the rate of spontaneous infection was higher compared to rubella infection.^10^ Hence, protection against mumps is very pivotal.

Mumps can be transmitted by respiratory droplets, direct contact, or fomites. It can be easily transmitted to those living near the infected. In countries like ours, where there are no strict guidelines regarding isolation of those infected with the contagious disease. Many school children might be attending school resulting in the rapid spread among the schoolmates. Furthermore, because of poor documentation and lack of published studies, mumps is considered as an insignificant public health concern. However, mumps are widely prevalent in Nepal. We need to protect our citizens from this highly contagious disease. This can be done by incorporating the two-dose vaccination against mumps in the national immunization schedule.

## EFFICACY OF VACCINE, COST, AND A GLOBAL EXPERIENCE

The mumps vaccine is routinely administered in 123 WHO state members as of the end of 2022.^11^ There has been a decline in the incidence of mumps with universal vaccination.^3,5,12^ Mumps incidence has reduced by more than 88% in countries adopting single dose schedule whereas reduction is more than 97% in countries adopting 2 dose schedule.^5^ Similarly, many countries have achieved a target of <1 mumps case per 100,000 population.^13^ Finland became the first country to declare itself mumps-free after a national 2-dose MMR vaccination program for children.^14^

Various studies done in the USA, Sweden, and China have shown a greater incidence of encephalitis with the mumps virus being the culprit in the pre-vaccination era with the decline following the introduction of vaccination.^15,16^

Studies done in different countries have shown that the introduction of the mumps vaccine is economically justifiable when compared with the total costs of OPD visits, hospital admissions, morbidity, and complications management.^17-19^ It also needs to be stressed that no additional logistics will be required to incorporate the mumps vaccine in already existing measles and rubella vaccinations.

The Indian Academy of Pediatrics, committee on vaccination strongly recommends the use of the MMR vaccine instead of the MR vaccine in the universal immunization program as it believes mumps is a serious health concern in India.^20^ Nepal Pediatric Society is also advocating for the incorporation of mumps in the currently available measles-rubella (MR) vaccination in a national schedule.

## WAY FORWARD

As it is evident that the prevalence of mumps is increasing in Nepal with the expected rise in mumps-associated complications; a serious focus should be on the prevention of mumps infection. As vaccination against mumps is highly efficient and is also economical, it seems wise to introduce MMR (mumps, measles, rubella) instead of MR in the national immunization program of Nepal.
